# Right aortic arch with aberrant left innominate artery arising from Kommerell's diverticulum*

**DOI:** 10.1590/0100-3984.2013.1934

**Published:** 2016

**Authors:** Ângela Faistauer, Felipe Soares Torres, Carlo Sasso Faccin

**Affiliations:** 1 MD, Radiologist at the Hospital Escola da Universidade Federal de Pelotas (UFPel), Pelotas, RS, Brazil.; 2 PhD, MD, Radiologist in the Radiology Department of the Hospital de Clínicas de Porto Alegre (HCPA), Porto Alegre, RS, Brazil.; 3 MD, Radiologist at the Hospital de Clínicas de Porto Alegre (HCPA), Porto Alegre, RS, Brazil.

**Keywords:** Right aortic arch, Left innominate artery, Kommerell's diverticulum, Arco aórtico à direita, Artéria inominada esquerda, Divertículo de Kommerell

## Abstract

We report a case of an uncommon thoracic aorta anomaly-right aortic arch with aberrant left innominate artery arising from Kommerell's diverticulum-that went undiagnosed until adulthood.

## Abstract (pt)

É apresentado o caso de uma rara anomalia da aorta torácica - arco aórtico à direita associado a artéria inominada esquerda aberrante com origem em divertículo de Kommerell -, com diagnóstico apenas na vida adulta.

## INTRODUCTION

Right aortic arch associated with aberrant left innominate artery is a rare congenital anomaly of the thoracic aorta, caused by a defect in the mechanism of regression of the branchial arches during embryogenesis.

Right aortic arch associated with aberrant left subclavian artery secondary to Kommerell's diverticulum is a common vascular anomaly, numerous cases having been reported^(1)^. However, there have been only 12 reports of right aortic arch associated with aberrant left innominate artery in the international literature, with no cases reported in Brazil.

## CASE REPORT

A 48-year-old female sought treatment for dyspnea and mild dysphagia that she had had since childhood, with progressive worsening of dyspnea in the last year. In the initial clinical evaluation, the patient reported dyspnea on mild exertion and mild discomfort when swallowing, without any other symptoms. She reported that she had no history of smoking, lung disease, or cardiovascular disease. The physical examination revealed an enlarged thyroid gland but was otherwise unremarkable.

**Figure 1:**
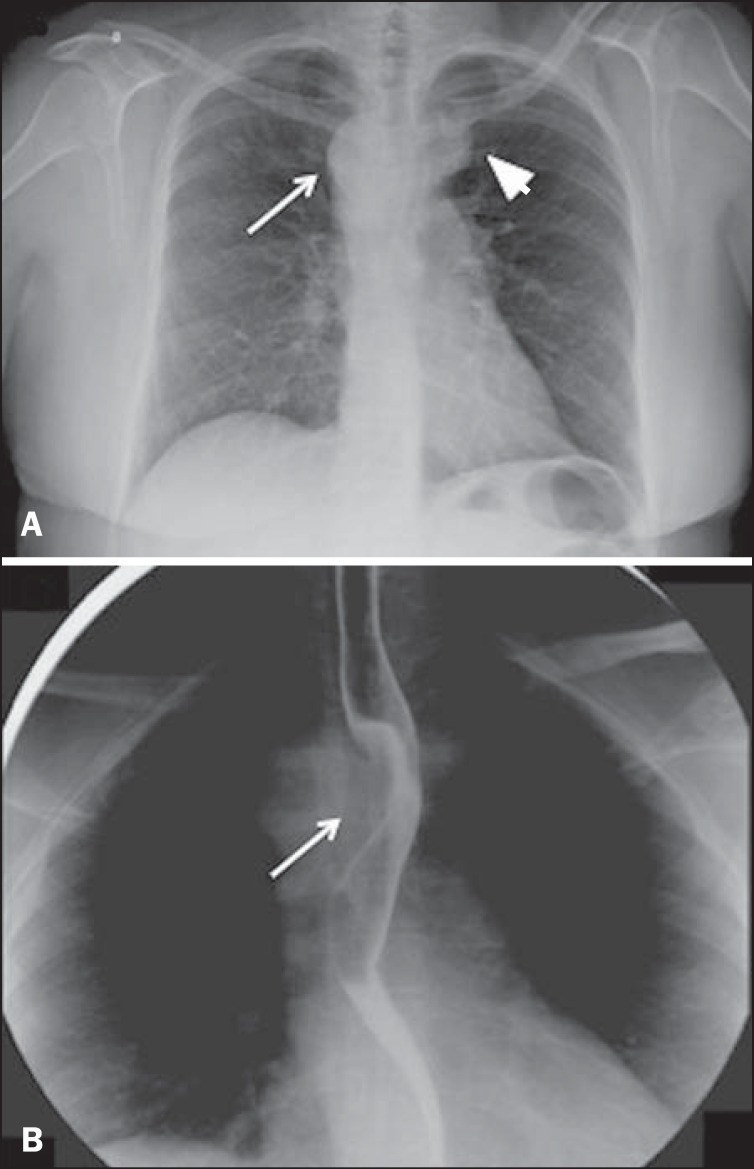
**A:** Posteroanterior chest X-ray showing right aortic arch (arrow) and widening of the superior mediastinum on the left (arrowhead), probably due to vascular ectasia. **B:** Contrast-enhanced radiographic study of the esophagus showing extrinsic compression of the middle third of the esophagus (arrow), at the level of the aortic arch.

A chest X-ray showed a right aortic arch and widening of the superior mediastinum (Figure 1A). Contrast-enhanced radiographic examination of the esophagus showed extrinsic compression of the proximal esophagus, at the level of the aortic arch (Figure 1B).

**Figure 2:**
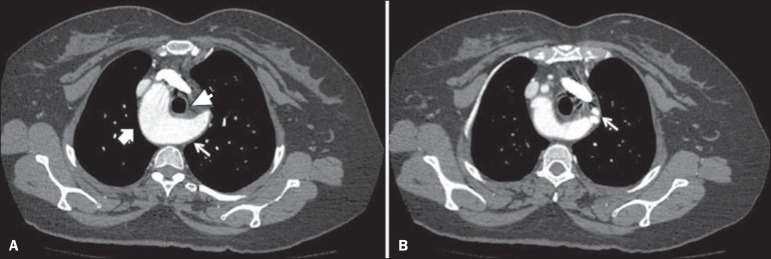
Computed tomography angiography of the chest, in the axial plane. **A:** Right aortic arch (thick arrow), identifying Kommerell's diverticulum on the left (thin arrow), resulting in anterior displacement of the esophagus (arrowhead). **B:** Image with a section that is more cranial than that in **A**, showing Kommerell's diverticulum that gives rise to the left common carotid and subclavian arteries (arrow).

**Figure 3:**
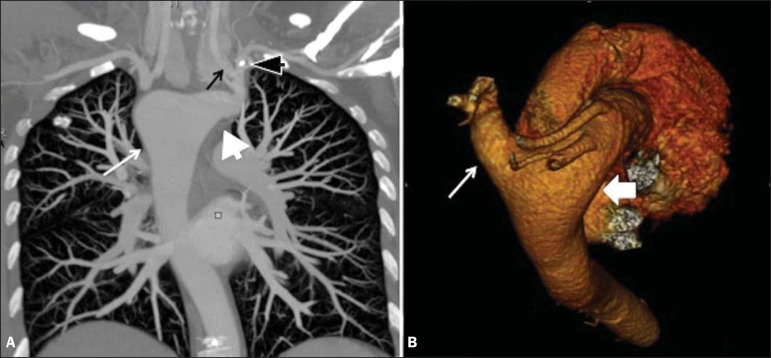
**A:** Chest computed tomography angiography, maximum intensity projection reconstruction, in the coronal plane, showing right aortic arch (thin white arrow) and Kommerell's diverticulum (white arrowhead), giving rise to the left common carotid artery (thin black arrow) and left subclavian artery (black arrowhead). **B:** Threedimensional reconstruction with superoposterior view of the aorta and great vessels showing the aortic arch on the right (thick arrow) with aberrant left innominate artery originating from Kommerell's diverticulum (thin arrow).

Computed tomography angiography of the chest (Figures 2 and 3A) with three-dimensional reconstructions (Figure 3B) showed right aortic arch with left innominate artery, the proximal segment of which was dilated (Kommerell's diverticulum), with a posteror trajectory into and compressive effect on the esophagus. A multinodular substernal goiter, causing a slight reduction in the diameter of the trachea, was also identified. The patient underwent total thyroidectomy, with favorable postoperative evolution, opting for clinical follow-up of the vascular malformation.

## DISCUSSION

Vascular diseases of the chest have been the subject of recent publications in the radiology literature of Brazil^(2-4)^. Malformations of the aorta account for 15-20% of congenital heart defects. Such anomalies can be diagnosed when there are symptoms of compression of the airway or esophagus, or incidentally on imaging studies performed for any of a number of causes. According to autopsy studies^(5,6)^, right aortic arch affects 0.04-0.1% of the population.

One of the major explanations for anomalies of the aortic arch is given by the Rathke diagram, which consists of six paired branchial arches along the anterior wall of the primitive gut, connecting the dorsal and ventral aspects of the aorta. The final formation of the aortic arch and its branches occurs after migration and regression of those arches, around the seventh week of pregnancy, and its malformations are due to defects in that mechanism. Right aortic arch results from the dissolution of the left, rather than the right, dorsal aortic root. This anomaly can be acompanied by esophageal atresia and tracheoesophageal fistula^(6,7)^.

The best known classification for right aortic arch consists of three subgroups: right aortic arch with mirror image branches, which has a strong association with congenital heart disease; right aortic arch with aberrant left subclavian artery, which can arise as the last branch of the right aortic arch or as a remnant of the right dorsal aortic arch, known as Kommerell's diverticulum^(1,5,6)^; and right aortic arch with isolated left subclavian artery.

The malformation of the aortic arch described in the case reported here (right aortic arch with aberrant left innominate artery associated with Kommerell's diverticulum) is extremely rare, diverging from the classical descriptions of aortic arch anomalies. That is borne out by the small number of cases reported in the literature-only 12 since its first description in 1968^(8)^.

Right aortic arch with aberrant left innominate artery is an anomaly similar to right aortic arch with aberrant left subclavian artery and retroesophageal (Kommerell's) diverticulum. In the former, the innominate artery usually originates from a diverticulum that also gives rise to the ductus arteriosus (or ligamentum arteriosum) on the left, which communicates with the proximal portion of the left pulmonary artery, completing a vascular ring^(9)^.

In patients with the malformation described here, the symptoms include compression of the trachea or the esophagus, dyspnea, and dysphagia, all of which typically manifest in childhood^(7)^. In our case, the diagnosis was made in adulthood, as in the case reported by the Midiri et al.^(7)^. In addition, our patient showed clinical symptoms that prompted imaging studies, through which the vascular malformation was diagnosed, but were not necessarily related to this anomaly, given the concomitant voluminous substernal goiter.

The vascular abnormalities of the aortic arch can be diagnosed by various imaging modalities, including chest X-ray, bronchoscopy, barium swallow, echocardiography, magnetic resonance, and computed tomography angiography. In addition to those techniques, surgical procedures can provide details of the anatomical aspects^(8)^.

The case reported here corroborates evidence in the literature of how the volumetric post-processing of computed tomography angiography images can facilitate the assessment of vascular changes in the thoracic aorta and add data on the effect that such abnormalities have on the adjacent mediastinal structures^(10)^.
